# Assessment of Heavy Metal Pollution in Mangrove Sediments of Liusha Bay, Leizhou Peninsula, China

**DOI:** 10.3390/toxics13110961

**Published:** 2025-11-06

**Authors:** Xianhui Yang, Huamei Huang, Ping Hu, Hong Luan, Bei Song, Zhaoyong Zheng, Cuiping Zhang, Ran Yan, Kang Li

**Affiliations:** 1 Technology Innovation Center for South China Sea Remote Sensing, Surveying and Mapping Collaborative Application, Ministry of Natural Resources, Guangzhou 510300, China; yangxianhuia@163.com (X.Y.);; 2 South China Sea Development Research Institute, Ministry of Natural Resources, Guangzhou 510300, China; 3 South China Sea Institute of Oceanology, Chinese Academy of Sciences, Guangzhou 510301, China; 4 South China Sea Island Center, Ministry of Natural Resources, Guangzhou 510300, China; 5 School of Ocean Sciences, China University of Geosciences, Beijing 100083, China; 6 Shenzhen Port Group Co., Ltd., Shenzhen 518081, China

**Keywords:** Liusha Bay, mangrove forests, heavy metal pollution, ecological risk assessment, material source

## Abstract

Heavy metal pollution threatens coastal ecosystems. Mangrove sediments, as transitional zones, are prone to contaminant accumulation. This study investigated eight heavy metals (Cu, Pb, Ni, As, Cr, Zn, Cd, Co) in Liusha Bay (Leizhou Peninsula, China). Field sampling, lab analysis, and multivariate statistics were used to assess pollution sources and ecological risks. The results show Al and Fe dominate sediment composition, with elevated P, Mn, and Sr. Arsenic (As) exhibiting the highest pollution severity (50% sites moderately contaminated by I_geo_). Enrichment factors (EF) indicate anthropogenic contributions to As, Cu, Ni, and Co, while Cd and Pb originate mainly from natural sources. Ecological risk assessments highlight moderate risks for As and Cd at some sites. Source analysis identifies three dominant pathways: (1) lithogenic inputs (volcanic rock weathering) contributing Fe, Zn, Cr, and Ni; (2) biogenic materials (calcium carbonate-secreting organisms) influencing Cu, Mn, and Cd; and (3) anthropogenic activities (aquaculture, maritime traffic) linked to Cu and Pb. This study emphasizes localized monitoring of As and Cd in mangroves and calls for the integrated management of natural and anthropogenic drivers to mitigate pollution risks.

## 1. Introduction

Mangroves are salt-tolerant evergreen plant communities, serving as an integral part of marine ecosystems and a natural barrier against waves, soil erosion, floods, storms, and tsunamis [1,2,3]. With the rapid advancement of industrialization and urbanization, mangrove sediments have become important sinks for heavy metal pollutants [4]. These sediments can accumulate large amounts of heavy metals through physicochemical processes such as surface adsorption, ion exchange, and flocculation [5]. Heavy metals deposited in mangrove wetlands feature wide sources, a long retention time, and high remediation difficulty after pollution. They not only pose potential threats to ecosystems but also are hardly biodegradable once entering the ecological cycle—usually entering the food chain via lower organisms and water, disrupting the normal physiological metabolism of organisms, and ultimately endangering human health and the ecological environment through bioaccumulation [6,7].

Liusha Bay, located in southwestern Leizhou Peninsula, China, is a renowned marine pearl cultivation and production base, whose annual output accounts for ~70% of China’s total marine pearl production [8,9]. It harbors the largest seagrass beds in southern China’s coastal areas, along with mangroves and coral reefs, possessing high marine ecological research value [10]. Current studies on Liusha Bay mainly focus on seagrass bed distribution and ecological value [11], annual variations in epiphytes and their impacts on seagrass physiology [12], heavy metal enrichment and ecological risks in seagrass beds [13], marine environmental quality assessment [14], mangrove–seagrass bed ecological connectivity and carbon storage [10], heavy metal pollution of *Sardinella albella* [9], and bacterial communities on pearl culture facilities [8]. Liusha Bay’s mangroves belong to Zhanjiang National Mangrove Nature Reserve, China’s largest mangrove reserve [15]. In recent years, with the expansion of mariculture and increased maritime shipping/operations in Liusha Bay, the sea area faces ecological threats from heavy metals and other pollutants [13,14]. However, studies on the geochemical characteristics and heavy metal pollution of mangrove sediments in Liusha Bay remain unreported.

As a semi-enclosed tropical bay located on the southwestern coast of the Leizhou Peninsula in China, Liusha Bay supports a thriving mangrove ecosystem and serves as a key mariculture base for pearl oyster larvae, underscoring its significant ecological and economic value. Within this context, the present study aims to: (i) examine the concentration characteristics and spatial distribution of major elements, trace elements, and eight typical heavy metals (Cu, Pb, Ni, As, Cr, Zn, Cd, Co) in sediments from mangrove habitats—including Na’ao Bay, the Tugong River Basin, and the Yingli Estuary—as well as from an unvegetated tidal flat; (ii) comprehensively evaluate the extent of heavy metal pollution and potential ecological risks in mangrove sediments using the geo-accumulation index (I_geo_), enrichment factor (EF), and potential ecological risk index ($E_{r}^{i}$), supplemented by analyses of heavy metal speciation; (iii) identify the sources and natural background levels of heavy metals in sediments through multivariate statistical methods—such as Pearson correlation analysis, hierarchical cluster analysis, and principal component analysis—while also considering regional geological background and anthropogenic influences (e.g., aquaculture and shipping activities). The findings are expected to provide a theoretical foundation for regional ecological conservation and environmental management.

## 2. Materials and Methods

### 2.1. Overview of the Study Area

**Figure 1:**
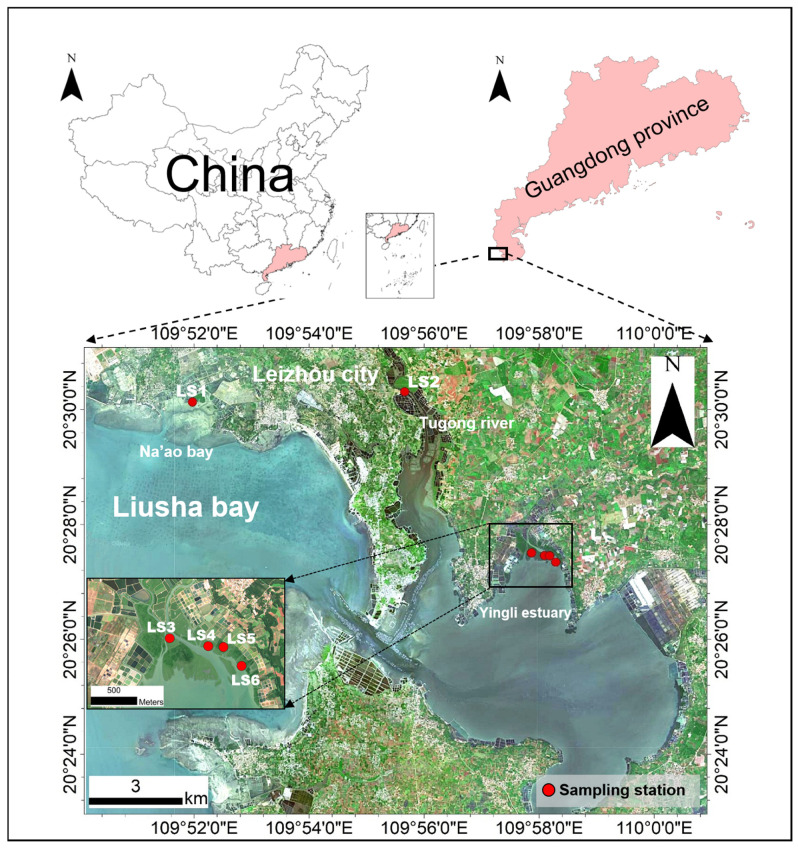
Study area and sampling station.

Liusha Bay is a semi-enclosed bay located in the northern South China Sea, by the southwestern Leizhou Peninsula [16] (Figure 1). A north–south-trending sand spit at the bay’s waist divides it into inner and outer bays: the inner bay is lagoon-shaped, connecting to the Beibu Gulf via a ~750 m wide channel through the sand spit, with the total water area of Liusha Bay being approximately 70 km^2^ [17]. There are mainly no large rivers inflowing into this area, which has a tropical monsoon climate with abundant sunlight and warm weather, providing superior conditions for aquaculture. The total area of marine pearl oyster seed cultivation reaches 13.6 km^2^ [18].

Liusha Bay boasts superior natural geographical conditions and rich biodiversity, with marine biological resources such as mangroves, seagrass beds, coral reefs, and various shellfish and fish [10,17]. Surveys show that its mangrove community is dominated by white mangrove (*Avicennia marina*), with scattered distributions of red mangrove (*Rhizophora stylosa*), black mangrove (*Aegiceras corniculatum*), and orange mangrove (*Bruguiera gymnorhiza*), covering a total area of over 230 hectares [10,19].

### 2.2. Sample Collection and Measurement

In July 2024, this study collected surface sediment (0–10 cm) samples from 6 stations in the mangrove area along Liusha Bay: 1 station in Na’ao Bay, 1 on the bank of the Tugong River, and 4 at the estuary of the Yingli River, with specific locations and distributions shown in Figure 1. Stations LS1–LS5 were set in contiguous mangrove growth areas, while LS6 was a mudflat site (tidal flat without mangrove coverage). These three areas largely represent the main distribution zones of mangroves in Liusha Bay. Given the extensive mangrove coverage and relatively high species diversity (including white mangrove, red mangrove, black mangrove, and orange mangrove) in the Yingli River estuary, three sampling sites were established in the mangrove area, along with one mudflat site.

At each sampling point, five sediment samples were collected within a 5 m × 5 m area. Then, a quarter of each of the five sediment samples was taken using the quartering method and mixed into one composite sample. Upon arriving at each sampling site, surface humus was first removed. Sediments at 0–10 cm depth were collected with a wooden shovel, sealed in clean polyethylene bags, and stored at 4 °C. Samples were naturally air-dried in the laboratory, crushed with wooden tools, and then ground in an agate mortar (plant residues were removed during grinding). The ground samples were passed through a 150-mesh nylon sieve for subsequent heavy metal content determination.

Element contents in sediments were measured by inductively coupled plasma mass spectrometry (ICP-MS) using Agilent 5110 and Agilent 7900 instruments (Agilent Technologies Inc., Santa Clara, CA, USA). The test adopted the simplified ISO 11466 method [20], with method errors within 10%, meeting analytical requirements. A blank sample, repeated sample, and certified reference material (GBM321-8; This standard substance is sourced from Geostats Pty Ltd., O’Connor, Australia) were inserted during the laboratory test to ensure data. Heavy metal speciation was analyzed via the Tessier five-step sequential extraction method to determine exchangeable, carbonate-bound, Fe-Mn oxide-bound, organic matter-bound, and residual fractions. All analyses were conducted by the ALS Laboratory in Guangzhou.

### 2.3. Contamination and Risk Assessment

Geo-accumulation index (I_geo_), Enrichment factor (EF) and Ecological risk assessment ($E_{r}^{i}$) were used to assess heavy metal pollution and ecological risk in sediments. The specific calculation process of these evaluation indicators is shown in Table 1.

### 2.4. Multivariable Statistical Analysis

Pearson’ correlation analysis is used to quantify the strength and direction of linear correlations between two continuous variables [27], which can determine the symbiotic relationships among heavy metals through correlation coefficients, laying a foundation for source identification. Hierarchical cluster analysis (HCA), a commonly used unsupervised machine learning method, realizes the gradual merging (or splitting) and classification of samples (or variables) by calculating their similarity or distance. It finally forms a dendrogram to intuitively present the hierarchical clustering process of data [28], facilitating the identification of heavy metal groups with similar source characteristics. Principal component analysis (PCA) aims to convert multiple interrelated variables into a few independent comprehensive indicators (i.e., principal components) while maximizing the retention of original data information (characterized by variance), thereby simplifying data dimensions and facilitating in-depth analysis of inherent data laws [29]. To clarify the main sources of 8 heavy metals in Liusha Bay mangrove sediments, this study combined Pearson correlation analysis, HCA, and PCA for source identification. All statistical analyses were conducted using Origin 2025b and SPSS (Statistical Package for the Social Sciences) 24 software.

## 3. Results

### 3.1. Concentration Characteristics of Sediment Elements

#### 3.1.1. Major Elements

**Figure A1:**
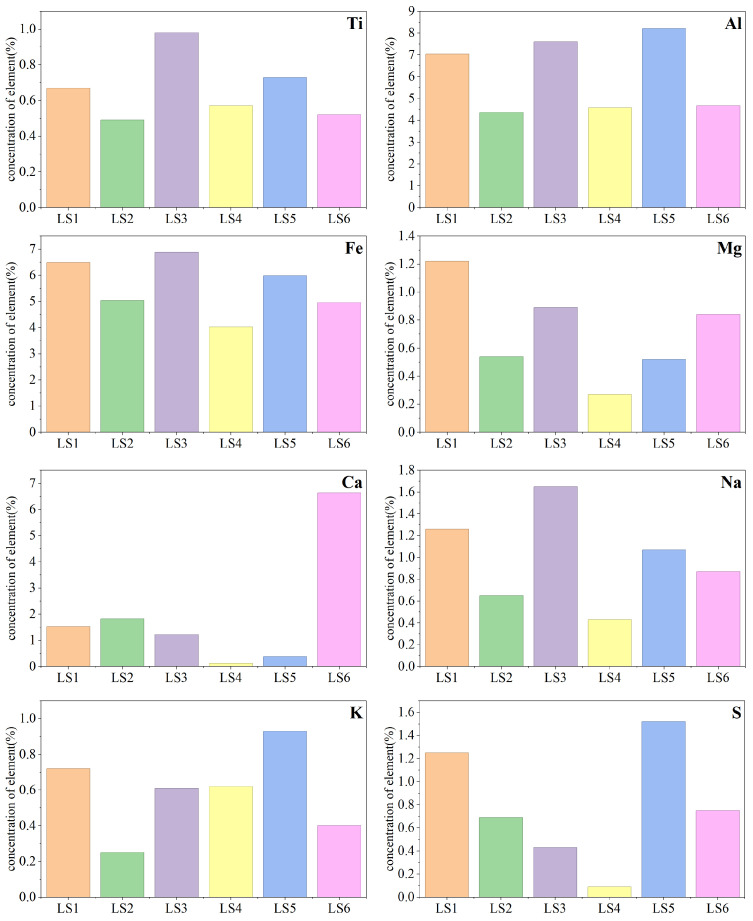
Concentrations of major elements in mangrove sediments of Liusha Bay.

Analysis results of the concentrations of eight major elements in mangrove sediments from Liusha Bay (Table 2, Figure A1) showed the following: the concentrations of Al and Fe were significantly higher, with Al slightly higher than Fe, and the average concentrations of both exceeded 5% (mass fraction); the concentration of Ca was also relatively high, with an average value close to 2%; the average concentrations of the remaining five major elements all ranged from 0.5% to 1%, sorted in descending order of concentration as Na > S > Mg > Ti > K.

In terms of concentration comparison between different stations, the concentrations of Al, Fe, Ti, and Na in Stations LS1, LS3, and LS5 were generally higher than those in Stations LS2, LS4, and LS6 (mudflat). The distribution of extreme concentrations showed distinct station-specificity: Station LS1 had the highest Mg concentration; Station LS3 had the highest concentrations of Ti, Fe, and Na; Station LS5 had the highest concentrations of Al, K, and S; Station LS6 had the highest Ca concentration; Station LS4 had the lowest concentrations of Fe, Mg, Ca, Na, and S; and Station LS2 had the lowest Al concentration.

#### 3.1.2. Trace Elements

**Figure A2:**
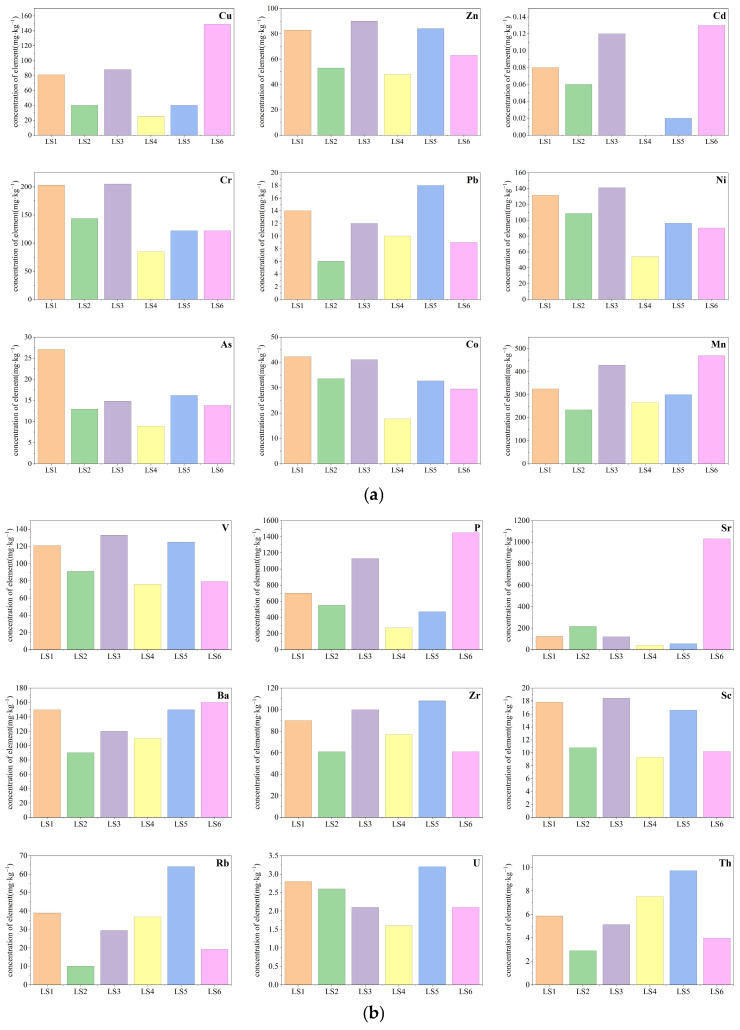
(**a**,**b**) Concentrations of trace elements in mangrove sediments of Liusha Bay.

Analysis results of the concentrations of 18 trace elements in the sediments (Table 3a,b; Figure A2a,b) indicated that the concentrations of different trace elements varied significantly. These elements can be classified into 6 grades based on average concentrations:

- High-concentration group (average concentration > 200 mg·kg^−1^). This group contained three elements: P, Mn, and Sr.
- Medium-high-concentration group (average concentration > 100 mg·kg^−1^). This group contained four elements: Cr, Ba, V, and Ni.
- Medium-concentration group (average concentration 70–83 mg·kg^−1^). This group contained three elements: Zr, Cu, and Zn. The concentrations of these three elements at most stations were below 100 mg·kg^−1^.
- Medium-low-concentration group (average concentration 32.8–33.1 mg·kg^−1^). This group contained two elements: Rb and Co, both with a concentration range of 10–65 mg·kg^−1^.
- Low-concentration group (average concentration 2.4–16 mg·kg^−1^). This group contained five elements. Among them, As, Sc, and Pb had an average concentration of 11–16 mg·kg^−1^ (concentration range 8–30 mg·kg^−1^); Th and U had an average concentration of 2.4–5.85 mg·kg^−1^ (concentration range 1–10 mg·kg^−1^).
- Lowest-concentration group. This group contained only Cd, with a concentration range of 0.02–0.13 mg·kg^−1^ and an average concentration of merely 0.08 mg·kg^−1^.

In terms of differences among stations: the concentrations of Zn, Pb, V, Zr, and Sc in Stations LS1, LS3, and LS5 were relatively higher, and the concentrations of Cr, Ni, and Co in Stations LS1 and LS3 were also at relatively high levels. The stations with the highest concentrations of each element were as follows: Station LS1 had the highest concentrations of As and Co; Station LS3 had the highest concentrations of Zn, Cr, Ni, V, and Sc; Station LS5 had the highest concentrations of Pb, Zr, Rb, and U. The concentrations of Cu, Zn, As, Mn, P, and Ba in Stations LS2 and LS4 were relatively lower. Among these, Station LS2 had the lowest concentrations of Pb, Mn, Ba, Zr, Rb, and Th; Station LS4 had the lowest concentrations of Cu, Zn, Cd, Cr, Ni, As, Co, V, P, Sr, Sc, and U. The special station LS6 (the mudflat) had the highest concentrations of Cu, Cd, Mn, P, Sr, and Ba among all stations.

**Table 3:** Concentration of trace elements in sediments (mg·kg^−1^).

(**a**)									
**Station**	**Cu**	**Zn**	**Cd**	**Cr**	**Pb**	**Ni**	**As**	**Co**	**Mn**
LS1	81	83	0.08	203	14	131.5	27.1	42.3	325
LS2	40	53	0.06	144	6	108.5	12.9	33.6	234
LS3	88	90	0.12	205	12	141	14.8	41.1	428
LS4	25	48	<0.02	85	10	54	8.9	17.7	266
LS5	40	84	0.02	122	18	96.2	16.2	32.7	300
LS6	149	63	0.13	122	9	90.3	13.8	29.5	469
Mean	71	70	0.08	147	11.5	103.6	15.6	32.8	337
S.D.	46	18	0.04	48	4	31.3	6.1	8.9	93
MAX	149	90	0.13	205	17.6	141	27.1	42.3	469
Min	25	48	0.02	85	6.2	54	8.9	17.7	234
upper-crust element concentration [23]	28	67	0.09	92	17	47	4.8	17.3	774
(**b**)									
**Station**	**V**	**P**	**Sr**	**Ba**	**Zr**	**Sc**	**Rb**	**U**	**Th**
LS1	121	700	123	150	90	17.8	38.9	2.8	5.86
LS2	91	550	216	90	61	10.8	10.1	2.6	2.9
LS3	133	1130	119	120	100	18.4	29.5	2.1	5.13
LS4	76	270	39	110	77	9.3	36.8	1.6	7.52
LS5	125	470	55	150	108	16.6	64.1	3.2	9.73
LS6	79	1450	1030	160	61	10.2	19.3	2.1	3.98
Mean	104	762	264	130	83	13.9	33.1	2.4	5.85
S.D.	25	444	381	28	20	4.2	18.7	0.6	2.47
MAX	133	1450	1030	160	108	18.4	64.1	3.2	9.73
Min	76	270	39	90	61	9.3	10.1	1.6	2.9
upper-crust element concentration [23]	97	655	320	628	193	14	84	2.7	10.5

#### 3.1.3. Spatial Distribution Patterns of Typical Heavy Metals

**Figure A3:**
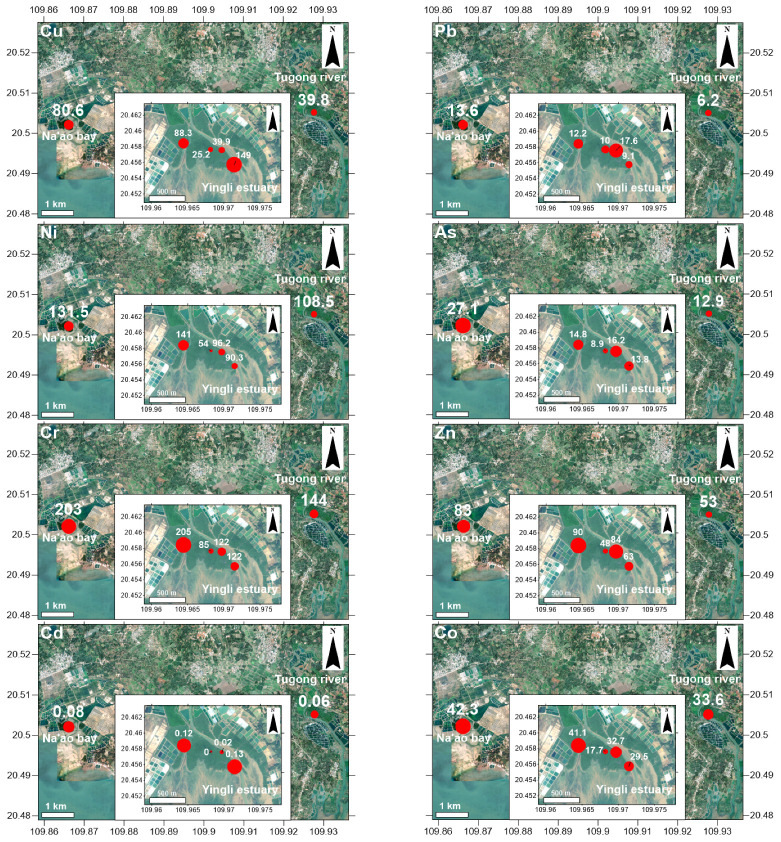
Spatial distribution of heavy metal concentrations in mangrove sediments of Liusha Bay. The red circles represent the sampling points, and the size of the red circles indicates the level of heavy metal concentration.

Figure A3 presents the spatial distribution characteristics of eight common heavy metals (Cu, Pb, Ni, As, Cr, Zn, Cd, and Co) in mangrove sediments from Liusha Bay. Combined with the division of survey areas (three typical mangrove distribution areas), the spatial differentiation patterns are as follows: for the Tugong River Basin (Station LS2), the overall heavy metal concentrations were relatively low; for Na’ao Bay (Station LS1), the overall concentrations were relatively high; and for the Yingli Estuary (Stations LS3–LS6), the spatial variation was significant—specifically, concentrations in Stations LS3, LS5, and LS6 (mudflat) were relatively high, while those in Station LS4 were relatively low.

### 3.2. Pollution Level and Ecological Risk Assessment

#### 3.2.1. Geo-Accumulation Index (I_geo_)

**Figure 2:**
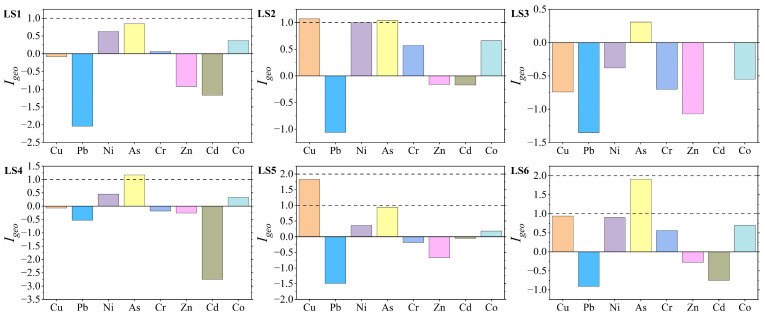
Evaluation results of the geo-accumulation index. Different pollution grades are divided using dashed lines.

**Figure A4:**
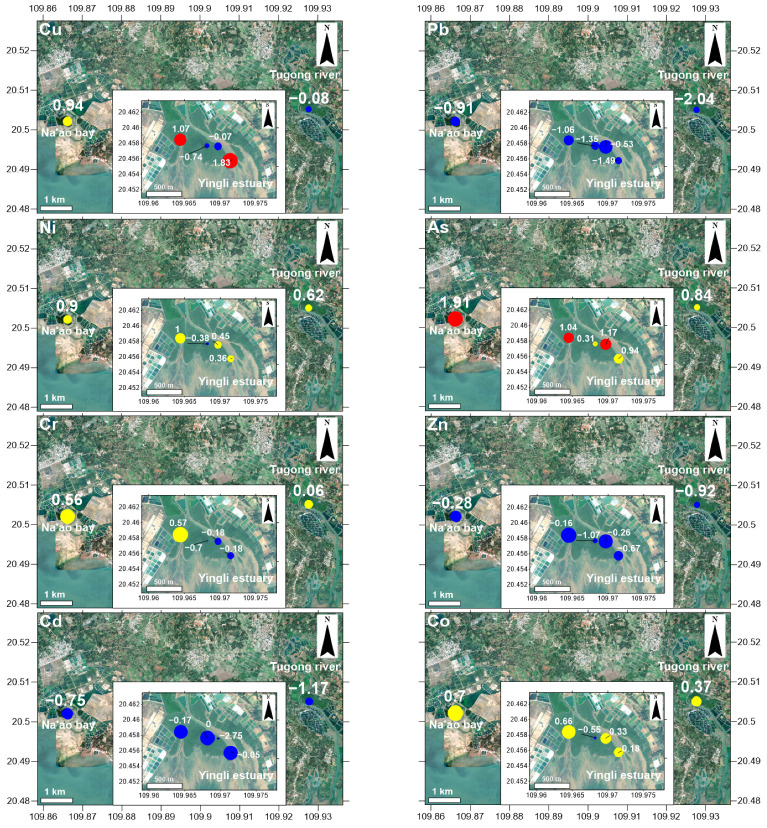
Spatial distribution of geo-accumulation index of mangrove sediments in Liusha Bay. According to the degree of pollution from light to severe, blue circles represent “Uncontaminated”, yellow circles represent “Slightly”, and red circles represent “Moderately”.

The index of geo-accumulation (I_geo_) was used to evaluate the accumulation characteristics and pollution levels of eight heavy metals (Cu, Pb, Ni, As, Cr, Zn, Cd, and Co) in mangrove sediments from Liusha Bay (Figure 2 and Figure A4). The results showed that the I_geo_ ranges of the eight heavy metals were as follows: Cu −0.74~1.83, Pb −2.04~−0.53, Ni −0.38~1.00, As 0.31~1.91, Cr −0.70~0.57, Zn −1.07~−0.16, Cd −2.75~0.00, and Co −0.55~0.70.

The average I_geo_ values of the eight heavy metals ranged from −1.23 to 1.04, and the accumulation degree sorted by average values was in the order of As > Cu > Ni > Co > Cr > Zn > Cd > Pb. There were significant differences in I_geo_ values among different stations: Station LS1 had the highest I_geo_ values for As and Co; Station LS3 had the highest I_geo_ values for Ni and Cr; Station LS6 had the highest I_geo_ value for Cu. In contrast, Station LS4 had relatively low I_geo_ values for all eight heavy metals, while Stations LS1 and LS3 generally had relatively high I_geo_ values for all heavy metals.

The pollution levels classified by element are as follows:

- Copper (Cu): Covered three levels, i.e., “uncontaminated”, “slightly”, and “moderately”, accounting for 50%, 16.7%, and 33.3% of the total stations, respectively. This indicates that Cu contamination existed in the study area, and 33.3% of the stations had reached the moderately contaminated level.
- Lead (Pb), Zinc (Zn), and Cadmium (Cd): All stations were at the “uncontaminated” level, indicating that the sediments were not contaminated by Pb, Zn, or Cd.
- Nickel (Ni), Chromium (Cr), and Cobalt (Co): All included two levels, “uncontaminated” and “slightly”. Among them, half of the stations were “uncontaminated” for Cr (with relatively low pollution levels), while 83.3% of the stations were “slightly” for Co and Ni (with relatively high pollution levels).
- Arsenic (As) had the most severe contamination, with 50% of the stations at the “slightly” level and 50% at the “moderately” level. Arsenic contamination existed in all stations, with equal proportions of light and moderate contamination.

#### 3.2.2. Enrichment Factor (EF)

**Figure 3:**
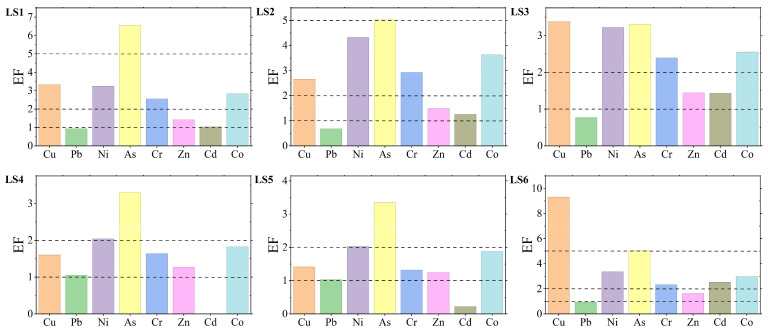
Evaluation results of enrichment factors. Different pollution grades are divided using dashed lines.

**Figure A5:**
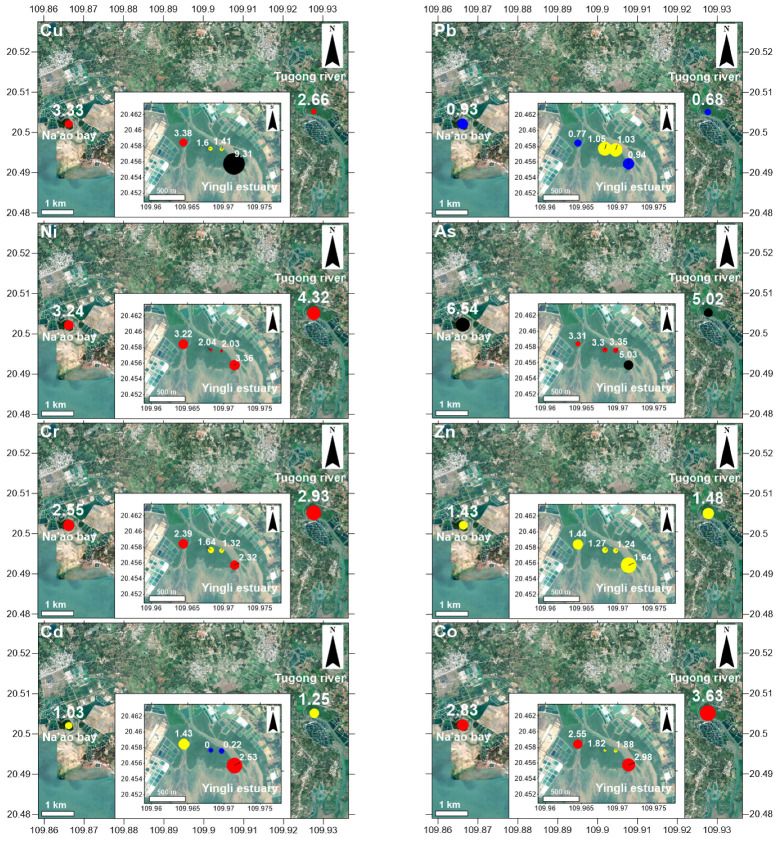
Spatial distribution of enrichment factors of mangrove sediments in Liusha Bay. According to the degree of pollution from light to heavy, blue circles represent “Uncontaminated”, yellow circles represent “Slightly”, red circles represent “Moderately”, and black circles represent “Moderately/Heavily”.

The enrichment factor (EF) was used to evaluate the enrichment characteristics and pollution levels of eight heavy metals in mangrove sediments from Liusha Bay (Figure 3 and Figure A5). The results showed that the average EF values of the eight heavy metals, sorted in descending order, were: As (4.43) > Cu (3.62) > Ni (3.04) > Co (2.62) > Cr (2.19) > Zn (1.42) > Cd (1.08) > Pb (0.90).

In terms of comparison among different stations, Station LS1 had the highest EF value for As, corresponding to the “moderately/heavily” pollution level; Station LS2 had the highest EF values for Ni, Cr, and Co, all of which corresponded to the “moderately” pollution level; Station LS4 had the highest EF value for Pb, corresponding to the “slightly” pollution level; and Station LS6 had the highest EF values for Cu, Zn, and Cd, corresponding to the “moderately/heavily”, “slightly”, and “moderately” levels, respectively.

Based on the EF pollution classification criteria, the pollution characteristics of the eight heavy metals are as follows (sorted in descending order of average EF values as mentioned above):

- Arsenic (As): The most severely polluted, with half of the stations at the “moderately” level and the remaining half at the “moderately/heavily” level;
- Copper (Cu): Half of the stations at the “moderately” level, one-third at the “slightly” level, and only Station LS6 at the “moderately/heavily” level;
- Nickel (Ni): All stations had EF values corresponding to the “moderately” level;
- Cobalt (Co): Consistent with the pollution characteristics of Cr, with one-third of the stations at the “slightly” level and the remaining two-thirds at the “moderately” level;
- Chromium (Cr): Consistent with the pollution characteristics of Co, with one-third of the stations at the “slightly” level and the remaining two-thirds at the “moderately” level;
- Zinc (Zn): Relatively less polluted among the eight heavy metals, with all stations having EF values corresponding to the “slightly” level;
- Cadmium (Cd): Relatively less polluted among the eight heavy metals, with one-third of the stations at the “uncontaminated” level, half at the “slightly” level, and one-sixth at the “moderately” level;
- Lead (Pb): The least polluted, with two-thirds of the stations at the “uncontaminated” level and the remaining one-third at the “slightly” level.

#### 3.2.3. Ecological Risk Assessment

**Figure 4:**
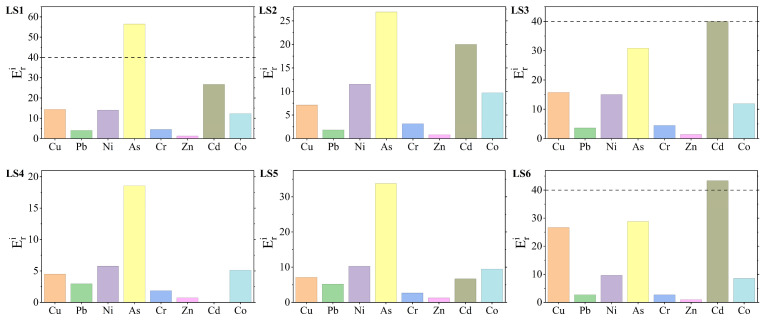
Results of ecological risk assessment. Different pollution grades are divided using dashed lines.

**Figure A6:**
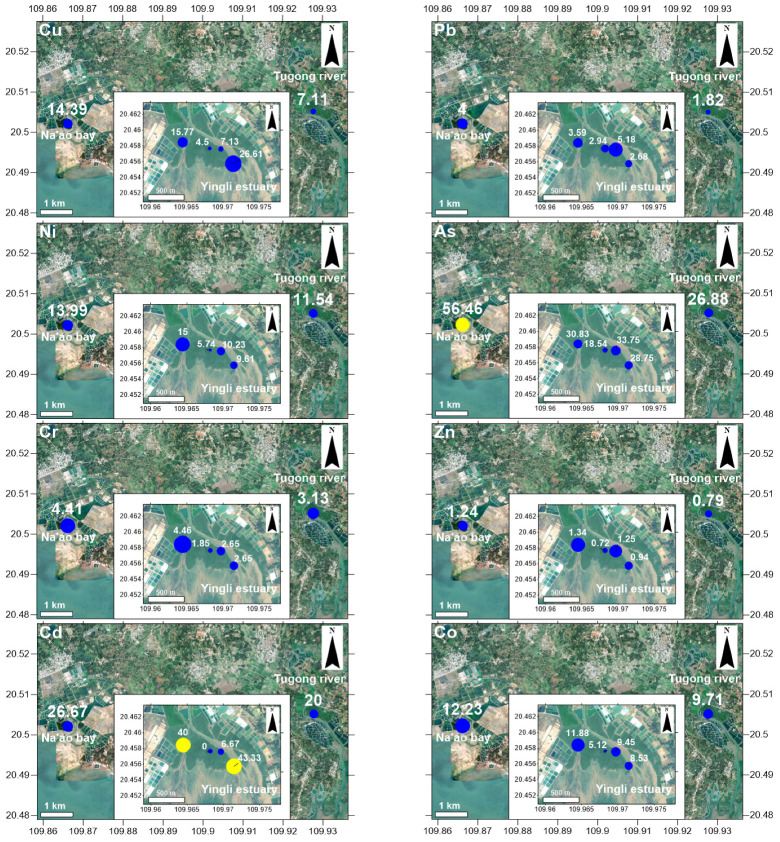
Spatial distribution of ecological risk indexes of mangrove sediments in Liusha Bay. According to the degree of ecological risk from light to heavy, the blue circle represents ‘Low risk’, and the yellow circle represents ‘Moderate risk’.

The potential ecological risk index ($E_{r}^{i}$) was used to evaluate the ecological risk levels of eight heavy metals in mangrove sediments from Liusha Bay (Figure 4 and Figure A6). The results showed that the average $E_{r}^{i}$ values of the eight heavy metals, sorted in descending order, were As (32.54) > Cd (22.78) > Cu (12.59) > Ni (11.02) > Co (9.49) > Pb (3.37) > Cr (3.19) > Zn (1.05).

In terms of comparison among different stations, Station LS1 had the highest $E_{r}^{i}$ values for Ni, As, and Co, among which As was at the moderate ecological risk level, while Ni and Co were both at the low level; Station LS3 had the highest $E_{r}^{i}$ values for Cr and Zn, and both were at the low ecological risk level; Station LS5 had the highest $E_{r}^{i}$ value for Pb, which was at the low ecological risk level; Station LS6 had the highest $E_{r}^{i}$ values for Cu and Cd, among which Cd was at the moderate ecological risk level, and Cu was at the low level.

The ecological risk levels of the eight heavy metals were relatively low in most stations. Only As and Cd exhibited moderate ecological risks in one station and two stations, respectively. Specifically, the moderate ecological risk of As occurred at Station LS1, and the moderate ecological risk of Cd occurred at Stations LS3 and LS6.

### 3.3. Multivariable Statistical Analysis

#### 3.3.1. Pearson Correlation Analysis Heatmap

**Figure 5:**
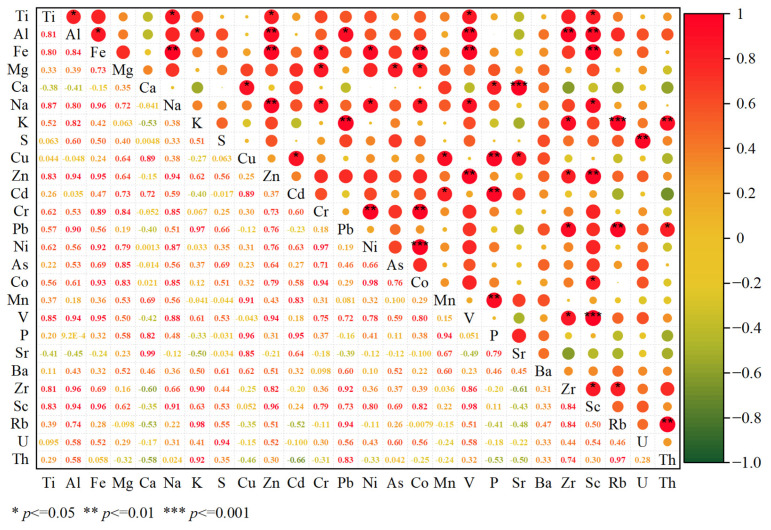
Pearson correlation heatmap of major and trace elements in mangrove sediments.

The Pearson correlation analysis results of major and trace elements in Liusha Bay mangrove sediments are shown in Figure 5. It was revealed that Cu was significantly correlated with Ca, Cd, Mn, Sr, and P; Pb with Al, K, Zr, Rb, and Th; Ni with Fe, Na, Cr, and Co; As with Mg; Cr with Na, Mg, Fe, Ni, and Co; Zn with Na, Fe, Al, Ti, V, Zr, and Sc; Cd with Cu, Mn, and P; and Co with Ni, Cr, Na, Mg, Fe, and Sc.

#### 3.3.2. Cluster Analysis (CA)

**Figure 6:**
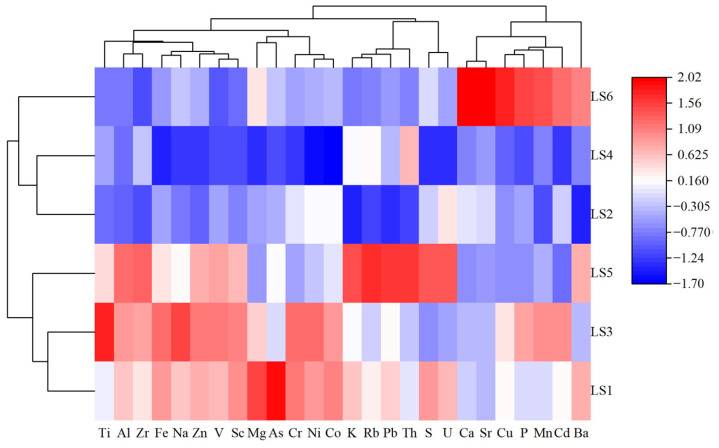
Heat map of cluster analysis of major and trace elements in mangrove sediments and sampling stations.

The heatmap of the cluster analysis for major elements, trace elements, and sampling stations in mangrove sediments from Liusha Bay is shown in Figure 6.

The cluster analysis of major and trace elements in the sediments indicated that all elements can be divided into three clusters: Cluster I (Ti, Fe, Na, Zn, V, Sc, Al, Zr, Mg, As, Cr, Ni, Co), Cluster II (K, Rb, Pb, Th, S, U), and Cluster III (Ca, Sr, Cu, P, Mn, Cd, Ba). Further subdivision revealed that Cluster I was split into two subclusters—Subcluster I_1_ (Ti, Fe, Na, Zn, V, Sc, Al, Zr) and Subcluster I_2_Mg, As, Cr, Ni, Co); Cluster II was split into two subclusters—Subcluster II_1_ (K, Rb, Pb, Th) and Subcluster II_2_ (S, U); and Cluster III was split into three subclusters—Subcluster III_1_ (Ca, Sr), Subcluster III_2_ (Cu, P, Mn, Cd), and Subcluster III_3_ (Ba only).

Cluster analysis of the sampling stations showed that all stations can be divided into 2 clusters: Cluster IV (LS2, LS4, LS6) and Cluster V (LS1, LS3, LS5). Further subdivision revealed that: Cluster IV was split into two subclusters—Subcluster IV_1_ (LS6) and Subcluster IV_2_ (LS2, LS4)—and Cluster V was split into two subclusters—Subcluster V_1_ (LS5) and Subcluster V_2_ (LS1, LS3).

#### 3.3.3. Principal Component Analysis (PCA)

**Figure 7:**
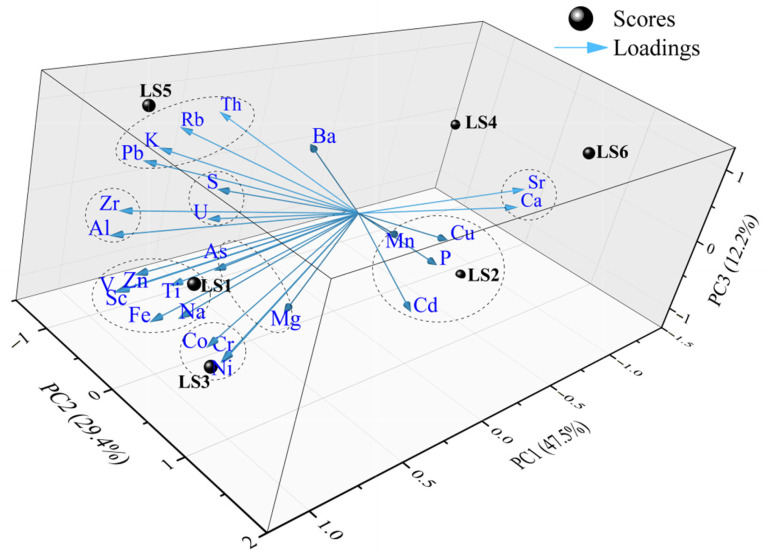
Load diagram of principal component analysis of major and trace elements in mangrove sediments in Liusha Bay. The circle dash line has circled the elements with similar characteristics.

The results of the principal component analysis (PCA) for major elements and trace elements in mangrove sediments from Liusha Bay are presented in Table 4 and Figure 7. A total of four principal components (PCs) were extracted, with a cumulative variance contribution rate of 97.448%, which can basically reflect the main sources of major elements, trace elements, and heavy metals in sediments of the study area:

- Principal Component 1 (PC1, variance contribution rate 47.521%) has high positive loadings on Zn, Sc, V, Fe, Al, Na, Zr, Co, Ti, Ni, Pb, Cr, As, Mg, K, S, U, and Rb;
- Principal Component 2 (PC2, variance contribution rate 29.437%) has high positive loadings on Cd, P, Cu, Ca, Sr, Mn, and Mg, and high negative loadings on Th, Rb, and K;
- Principal Component 3 (PC3, variance contribution rate 12.233%) has high positive loadings on Ba and Sr;
- Principal Component 4 (PC4, variance contribution rate 8.257%) has high positive loadings on U, S, and As.

Based on the loading plot of the first three principal components (Figure 7), it can be seen that most elements in mangrove sediments of Liusha Bay are significantly affected by PC1, while some elements (e.g., Cd, P, Cu) are obviously affected by PC2. Similarly to the results of the cluster analysis, there are nine obvious element clusters in the loading plot (Figure 7) (e.g., Sr and Ca, U and S, Al and Zr), and this phenomenon may indicate that these elements have similar material sources.

Furthermore, the score characteristics of each sampling station can also be observed from Figure 7: the scores of LS1, LS3, and LS5 are in the direction of positive loadings of PC1; correspondingly, the scores of LS2, LS4, and LS6 are in the direction of negative loadings of PC1; the scores of LS3 and LS6 are in the direction of positive loadings of PC2, while the scores of LS4 and LS5 are in the direction of negative loadings of PC2; the scores of LS5 and LS6 are in the direction of positive loadings of PC3, while the scores of LS2 and LS3 are in the direction of negative loadings of PC3.

**Table 4:** Factor analysis conducted for the mangrove sediments.

Variable	Load Factor			
	Factor 1	Factor 2	Factor 3	Factor 4
Zn	0.987	0.069	0.058	−0.118
Sc	0.986	−0.059	−0.148	−0.047
V	0.96	−0.128	−0.204	−0.065
Fe	0.958	0.188	−0.206	−0.017
Al	0.944	−0.272	0.093	−0.123
Na	0.91	0.302	−0.134	−0.248
Zr	0.823	−0.495	0.081	−0.264
Co	0.82	0.386	−0.338	0.244
Ti	0.784	−0.052	−0.207	−0.578
Ni	0.772	0.427	−0.451	0.114
Pb	0.77	−0.473	0.425	−0.053
Cr	0.757	0.41	−0.478	0.06
As	0.725	0.155	0.005	0.528
Mg	0.661	0.614	−0.033	0.238
K	0.649	−0.616	0.398	−0.127
S	0.623	−0.13	0.363	0.646
U	0.594	−0.219	0.084	0.677
Rb	0.533	−0.705	0.464	−0.055
Ba	0.477	0.235	0.823	0.124
Th	0.319	−0.807	0.465	−0.166
Mn	0.305	0.738	0.406	−0.444
Cd	0.291	0.947	−0.022	−0.123
P	0.194	0.911	0.248	−0.235
Cu	0.162	0.894	0.404	−0.089
Ca	−0.233	0.849	0.448	0.118
Sr	−0.304	0.784	0.521	0.062
Eigenvalue	12.355	7.654	3.181	2.147
Percentage of Variance (%)	47.521	29.437	12.233	8.257
Cumulative (%)	47.521	76.958	89.191	97.448

### 3.4. Heavy Metal Speciation Characteristics

**Figure 8:**
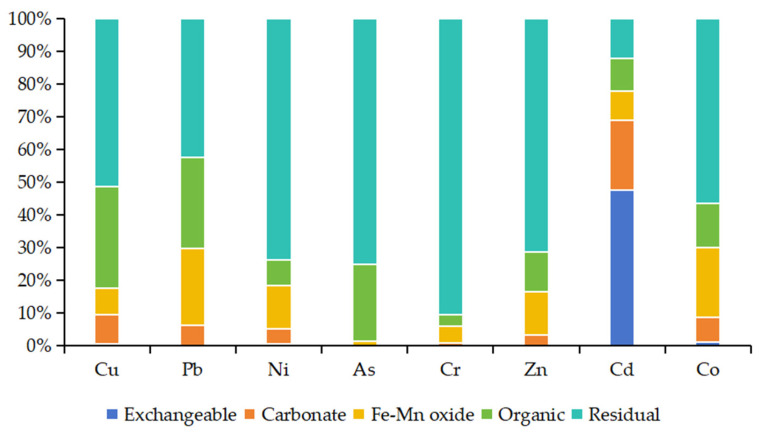
Sediment heavy metal sequential extraction in Liusha Bay.

The speciation distribution of heavy metals in mangrove sediments is shown in Figure 8. Except for Cd, the other seven heavy metals were mainly present in the residual fraction, which reduced their ecological risks: the residual fraction accounted for over 50% of Cu, Ni, As, Cr, Zn, and Co, and 42% of Pb.

Cd showed distinct speciation: its residual fraction accounted for only 12%, while the exchangeable fraction reached 47.5%. The organic fraction was the second dominant form for Cu, Pb, and As (23–31%), and the Fe-Mn oxide fraction was the second for Pb, Ni, Cr, and Zn (5–24%). In addition, no exchangeable fraction was detected for Pb and Zn, and no carbonate fraction for As.

Different elements had distinct dominant speciations: Ni had the highest concentrations in exchangeable and Fe-Mn oxide fractions, Cu in carbonate and organic fractions, and Cr in residual fraction. Cd was dominated by exchangeable and carbonate fractions, Pb by Fe-Mn oxide fraction, Cu by organic fraction, and Cr by residual fraction.

## 4. Discussion

### 4.1. Heavy Metal Pollution Comparison

#### 4.1.1. Comparison with Sediments from Other Regions in Liusha Bay

**Figure A7:**
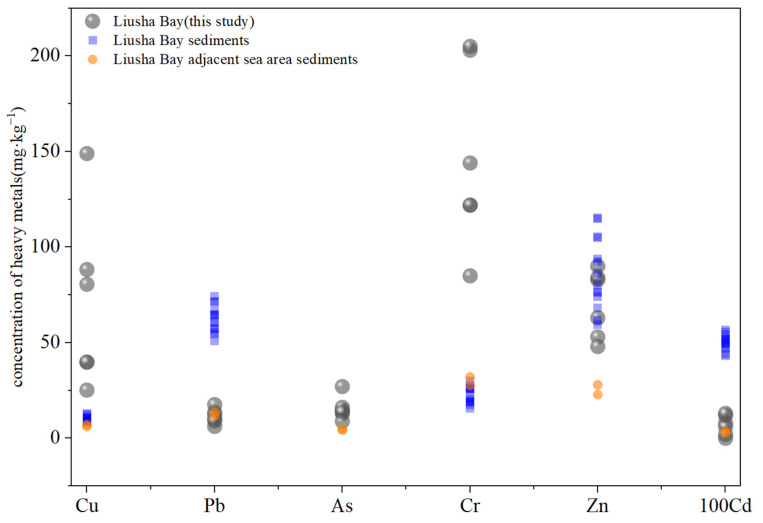
Comparison of heavy metal concentration in Liusha Bay. The data in the figure are sourced from References [17,31].

As shown in Table 5 and Figure A7, the concentrations of Cu and Cr in mangrove sediments of Liusha Bay are significantly higher than those in non-mangrove sediments inside the bay and offshore sediments outside the bay. Specifically, the average concentrations of Cu and Cr in mangrove sediments are 4–24 times and 5–11 times those in non-mangrove sediments inside the bay and offshore sediments, respectively; the concentration of As is slightly higher than that in the above two types of sediments; the concentration of Zn is slightly lower than that in non-mangrove sediments inside the bay but significantly higher than that in offshore sediments outside the bay; the concentrations of Cd and Pb are significantly lower than those in non-mangrove sediments inside the bay and are close to those in offshore sediments outside the bay.

**Table 5:** Comparison of heavy metal concentration in sediments of Liusha Bay.

Location	Cu	Pb	Ni	As	Cr	Zn	Cd	Co	Survey Time	Reference
Liusha Bay mangrove sediments (*n* = 6)	71	12	104	16	147	70	0.08	33	2024	This study
Liusha Bay sediments (*n* = 14)	10	62	-	-	23	87	0.5	-	2008	[17]
Liusha Bay shellfish aquaculture area sediments (*n* = 3)	17	24	-	10	23	72	0.19	-	2008~2009	[30]
Liusha Bay seagrass bed area sediments	3	2	7	-	14	17	0.03	-	2017~2018	[13]
Liusha Bay adjacent sea area sediments (*n* = 2)	7	13	-	5	30	25	0.03	-	2010	[31]

**Figure A8:**
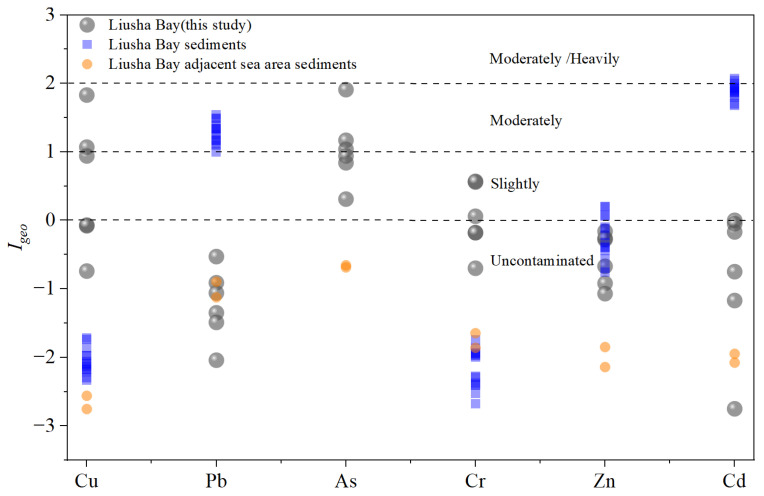
Comparison of geo-accumulation index of heavy metals in sediments of Liusha Bay. The data in the figure are sourced from References [17,31].

**Figure A9:**
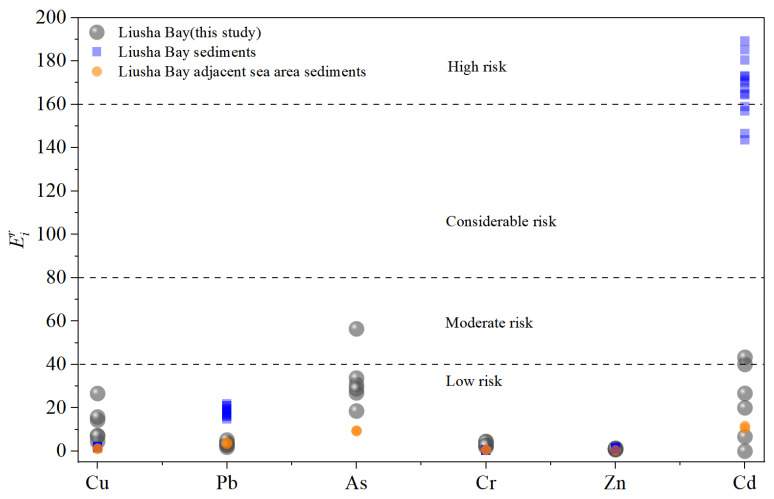
Comparison of ecological risk of heavy metals in sediments of Liusha Bay. The data in the figure are sourced from References [17,31].

In terms of heavy metal pollution levels (Figure A8 and Figure A9), compared with non-mangrove seabed sediments inside Liusha Bay, the index of geo-accumulation (I_geo_) of Cu in mangrove sediments is significantly higher, while there is no Cu pollution in non-mangrove sediments inside the bay; on the contrary, the I_geo_ of Pb in non-mangrove sediments inside the bay is significantly higher, and there is no Pb pollution in mangrove sediments or offshore sediments outside the bay; there is basically no Zn pollution in sediments across the entire Liusha Bay area (inside the bay, outside the bay, and mangroves); there is no Cd pollution in mangroves and offshore sediments outside the bay, while Cd pollution in non-mangrove sediments inside the bay is relatively severe.

In terms of ecological risk, the ecological risk level of Cd in non-mangrove sediments inside Liusha Bay reaches “high risk”; As and Cd levels in individual stations of mangrove sediments are at “moderate risk”; the ecological risk levels of other heavy metals in the entire area are relatively low, which may indicate that Cd in mangrove sediments is mainly derived from human activities such as aquaculture in Liusha Bay.

Previous studies have pointed out that tidal flat sediments and seabed sediments in bays usually have similar material sources [32], which is supported by the phenomenon in this study that the concentrations of As and Zn in sediments of the two regions are close; the differences in concentrations of other heavy metals are presumably due to different sedimentary environments and other pollution sources with variations. It can also be seen from Table 5, Table 6 and Table 7 that the concentration of Ni in mangrove sediments is higher than that in sediments of adjacent sea areas and mangroves in other regions. Combined with the characteristics of high Cu and Cr concentrations, it is inferred that mangrove sediments in this area may have received more volcanic rock weathering materials rich in Cu, Cr, and Ni. Previous studies have shown that Quaternary volcanic rocks are widely distributed in the southern part of the Leizhou Peninsula where Liusha Bay is located [33]. In addition, the high concentrations of Pb and Cd in non-mangrove seabed sediments inside Liusha Bay may be related to large-scale shellfish farming and shipping activities in the bay [30]—for example, aquaculture activities in the farming area of the Bay of Cádiz in southwestern Spain have significantly increased Pb content in sediments by 97% compared with non-farming areas [34].

From a temporal perspective, the sampling time of non-mangrove sediments inside Liusha Bay is 2008–2009 (over 10 years ago), and the sampling time of seagrass bed sediments is 2017–2018 (over 5 years ago). The high concentrations of Cu, Cr, and As in mangrove sediments may be related to the increased discharge of heavy metal-rich sewage due to the expansion of agricultural and industrial scales in this area over the past 10 years. Studies have shown that after 2008, the mariculture model in Liusha Bay shifted from being dominated by pearl shell farming to a pattern where cage fish farming, scallop hanging culture, and pearl shell farming coexist; farmers have added a large number of cages in the inner bay and the confluence of inner and outer bays, resulting in a weakened water exchange capacity between the inner and outer bays and a local deterioration of water quality [35]; by 2015, the aquaculture area in Liusha Bay had reached 16 km^2^, accounting for nearly 1/4 of its total area [36].

#### 4.1.2. Comparison with Mangrove Sediments in the Leizhou Peninsula

**Figure A10:**
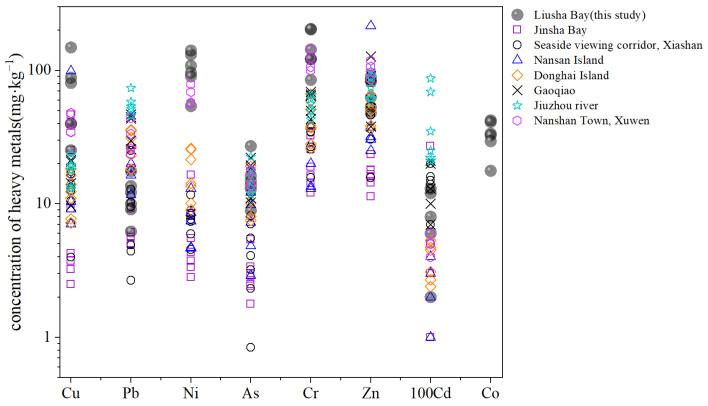
Comparison of heavy metal concentrations in mangrove sediments of Leizhou Peninsula. The data in the figure are sourced from References [15,37,38,39].

Compared with mangrove sediments in other regions of the Leizhou Peninsula (Table 6, Figure A10), the concentrations of Cu, Ni, and Cr in mangrove sediments of Liusha Bay are significantly higher, the concentration of As is slightly higher, the concentrations of Cd and Zn are at moderate levels, and the concentration of Pb is at a below-moderate level.

**Table 6:** Comparison of heavy metal concentrations in mangrove sediments of the Leizhou Peninsula.

Location	Cu	Pb	Ni	As	Cr	Zn	Cd	Co	Survey Time	Reference
Liusha Bay mangrove sediments (*n* = 6)	71	12	104	16	147	70	0.08	33	2024	This study
Jinsha Bay (*n* = 8)	12	9	7	4	26	26	0.06	-	2020	[15]
Seaside Viewing Corridor, Xiashan (*n* = 8)	12	10	8	4	31	46	0.13	-	2020	[15]
Nansan Island (*n* = 5)	28	19	8	7	22	68	0.03	-	-	[37]
Donghai Island (*n* = 7)	12	27	17	13	44	60	0.04	-	2017	[38]
Gaoqiao (*n* = 5)	17	38		16	59	81	0.14	-	2021	[15]
Jiuzhou river (*n* = 6)	18	55		16	54	79	0.43	-	2021	[15]
Nanshan Town, Xuwen (*n* = 5)	43	29	75	15	119	104	0.05	-	2017	[39]
Leizhou Peninsula mangrove sediments (*n* = 52)	14	21	16	10	37	51	0.06	7	2015~2016	[40]

**Figure A11:**
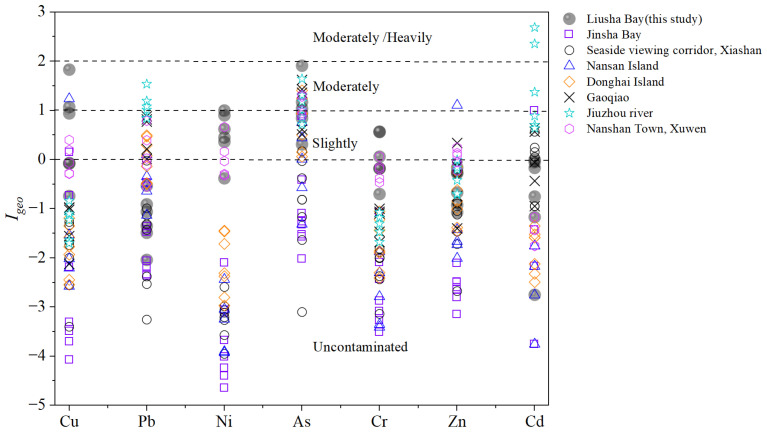
Comparison of geo-accumulation index of heavy metals in mangrove sediments of Leizhou Peninsula. The data in the figure are sourced from References [15,37,38,39].

**Figure A12:**
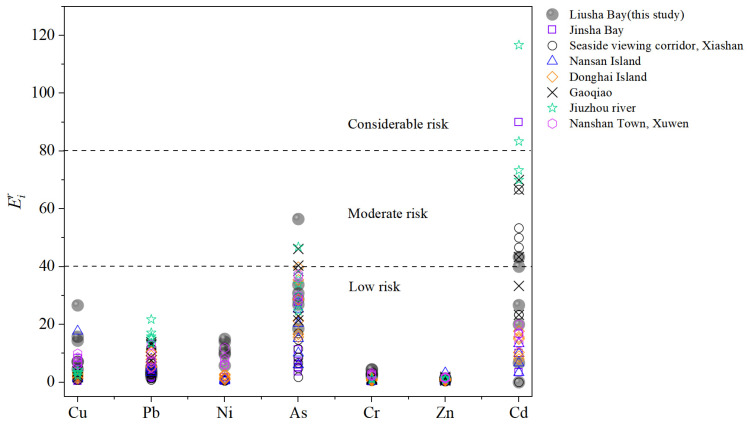
Comparison of ecological risk of heavy metals in mangrove sediments of Leizhou Peninsula. The data in the figure are sourced from References [15,37,38,39].

In terms of heavy metal pollution levels (Figure A11 and Figure A12), compared with mangrove sediments in other regions of the Leizhou Peninsula, the index of geo-accumulation (I_geo_) of Cu, Ni, and Cr in Liusha Bay’s mangrove sediments is at a relatively high level, with pollution levels ranging from unpolluted to moderately polluted, which is close to that of mangrove sediments in Nanshan Town, Xuwen (also located in the southern part of the Leizhou Peninsula); in contrast, Cu, Ni, and Cr in other regions are basically unpolluted. This difference may mainly stem from the extensive distribution of volcanic rock formations in the southern part of the Leizhou Peninsula [33], as weathered volcanic rocks can bring more Cu, Ni, and Cr elements.

The I_geo_ of Pb in Liusha Bay’s mangrove sediments is close to that in mangrove sediments of Jinsha Bay (northern Leizhou Peninsula) and the Seaside Viewing Corridor in Xiashan, all at the unpolluted level, while Pb pollution in other regions ranges from light to moderate. This may be mainly due to the developed industry and dense population in the northern Leizhou Peninsula, resulting in more Pb-containing industrial wastewater, domestic sewage, and automobile exhaust emissions [41].

In most mangrove sediment sampling stations across the Leizhou Peninsula, including Liusha Bay, the I_geo_ of Zn is less than 0, basically at the unpolluted level; only individual stations in Nanshan Town, Nansan Island, and Gaoqiao show Zn pollution ranging from light to moderate, which may be related to industrial wastewater and ship transportation wastewater in these regions [15,37,39].

The I_geo_ of As in Liusha Bay’s mangrove sediments is close to that in mangrove sediments of several northern regions of the Leizhou Peninsula, all at light to moderate pollution levels. As has diverse pollution sources; in addition to natural sources, industrial and agricultural activities may also generate As pollution [41]. Specifically, As pollution in Liusha Bay’s mangrove sediments may mainly come from aquaculture and agricultural activities in adjacent land areas, while As pollution in the mangrove sediments of the northern Leizhou Peninsula may mainly originate from industrial pollution [41,42].

In most mangrove sediment sampling stations across the Leizhou Peninsula, including Liusha Bay, Cd is at the unpolluted level; Cd pollution in the Jiuzhou River area ranges from light to moderate-severe, and individual stations in Jinsha Bay, Gaoqiao, and the Seaside Viewing Corridor show Cd pollution ranging from light to moderate. Cd pollution in these regions is mainly related to wastewater from aquaculture, agricultural planting, and mineral development, etc. [15].

#### 4.1.3. Comparison with Mangrove Sediments from Other Regions in China

**Figure A13:**
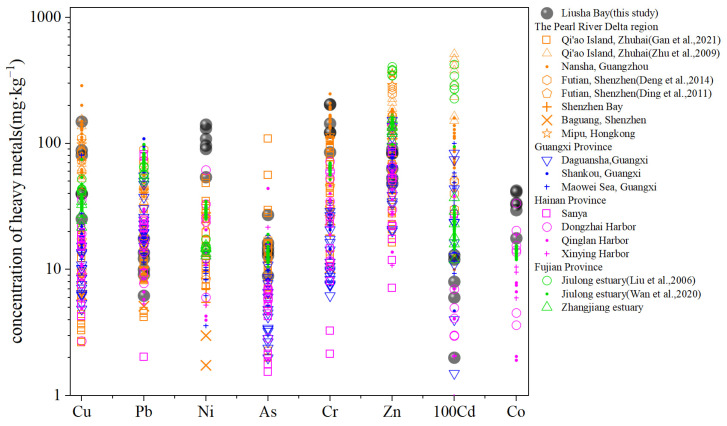
Comparison of heavy metal concentrations in mangrove sediments in China. The data in the figure are sourced from References [44,49,50,51,63,64,65,66,67,68].

The comparison results of heavy metal concentrations between mangrove sediments in Liusha Bay and those from other regions in China are presented in Table 7 and Figure A13. Among them, the Ni concentration in Liusha Bay’s mangrove sediments is significantly higher than that in other mangrove regions of China. Specifically, its average Ni concentration is 1.5–3.5 times that of mangrove sediments in the Pearl River Delta (including Guangzhou, Zhuhai, Shenzhen, Hong Kong, etc.), 3.9–7.1 times that in Hainan Province, 11.3–15.4 times that in the Guangxi Zhuang Autonomous Region, and 5.9–7.3 times that in Fujian Province.

**Table 7:** Comparison of heavy metal concentrations in mangrove sediments in China.

Location		Cu	Pb	Ni	As	Cr	Zn	Cd	Co	Survey Time	Reference
Liusha Bay mangrove sediments (*n* = 6)		71	12	104	16	147	70	0.08	33	2024	This study
The Pearl River Delta region	Nansha, Guangzhou (*n* = 6)	56	53	39	-	71	328	0.7	-	2021	[43]
	Haiou Island, Guangzhou (*n* = 6)	92	90	70	-	113	306	1.6	-	2021	[43]
	Qi’ao Island, Zhuhai (*n* = 6)	78	50	45	-	84	333	0.6	-	2021	[43]
	Futian, Shenzhen (*n* = 6)	80	52	30	-	58	158	0.4	-	2021	[43]
Hainan Province	Dongzhaigang (*n* = 3)	20	18	27	6	45	47	0.08	15	2009	[44]
	Qinglangang (*n* = 3)	21	18	15	16	31	39	0.17	7	2009	[44]
	Xinying Port (*n* = 3)	11	11	16	11	44	31	0.03	11	2009	[44]
	Bamen Bay (*n* = 6)	24	11	-	-	61	54	1.11	-	2014	[45]
	Lingshui (*n* = 16)	8	9	-	7	16	-	-	-	-	[46]
Guangxi Zhuang Autonomous Region	Beihai (*n* = 30)	9	21	7	15	-	27	0.22	2	2023	[47]
	Pearl Bay (*n* = 13)	11	14	7	7	24	28	0.07	-	2021	[48]
	Maowei Sea (*n* = 63)	25	18	9	12	30	60	0.34	-	2012	[49]
Fujian Province	Jiulong River Estuary (*n* = 5)	42	59	14	-	-	368	3.19	-	2003	[50]
	Zhangjiang Estuary (*n* = 6)	36	70	18	-	-	160	0.36	-	2004	[51]
Chinese mangrove sediments (*n* = 97)		23	76	-	8	18	86	0.21	-	2006~2007	[52]

**Figure A14:**
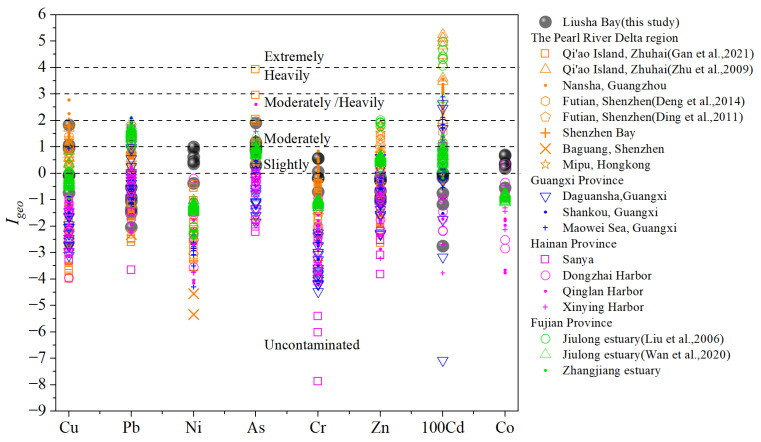
Comparison of geo-accumulation index of heavy metals in mangrove sediments in China. The data in the figure are sourced from References [44,49,50,51,63,64,65,66,67,68].

**Figure A15:**
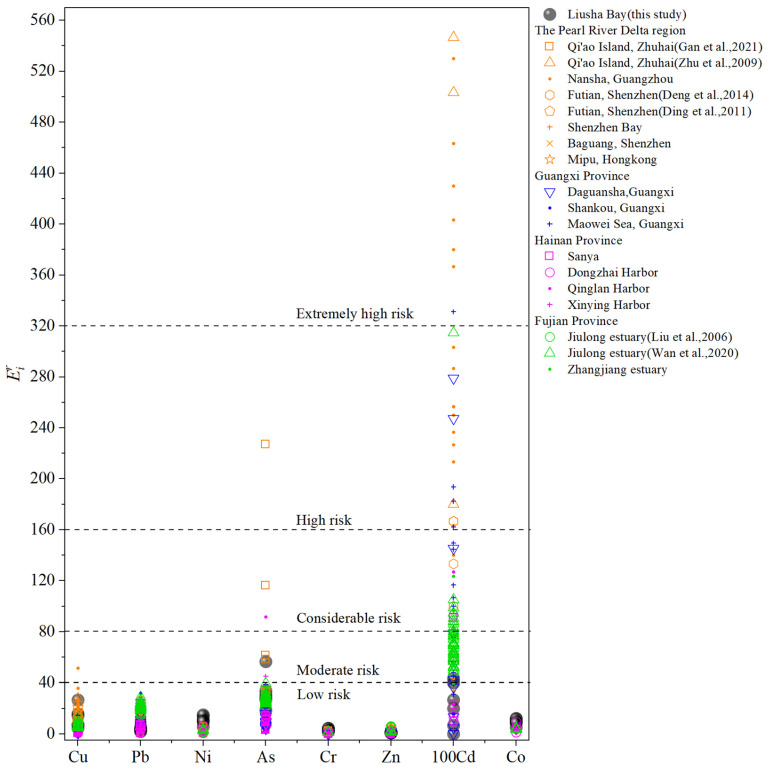
Comparison of ecological risk of heavy metals in mangrove sediments in China. The data in the figure are sourced from References [44,49,50,51,63,64,65,66,67,68].

In terms of heavy metal pollution levels (Figure A14 and Figure A15), compared with mangrove sediments from other regions in China, the I_geo_ values of Ni and Co in Liusha Bay’s mangrove sediments are significantly higher, basically at light to moderate pollution levels, while Ni and Co in mangrove sediments from other regions in China are basically unpolluted. In addition, the I_geo_ values of Cu, As, and Cr in Liusha Bay’s mangrove sediments are also relatively high, equivalent to those in mangrove sediments of the economically developed Pearl River Delta and Fujian Province (at light to moderate pollution levels); correspondingly, Cu, As, and Cr in mangrove sediments of the Guangxi Zhuang Autonomous Region and Hainan Province are basically unpolluted.

In terms of ecological risk, the overall ecological risk levels of Cu, Ni, Cr, and Co in sediments of China’s mangrove regions are relatively low; As in some stations of Liusha Bay is at the “moderate risk” level, As in Qinglan Harbor (Hainan Province) is at the “considerable risk” level, and As in some stations of Qi’ao Island (Zhuhai) is at the “high to extremely high risk” level.

Combined with the previous analysis results, the relatively high concentrations of Cu, Cr, Ni, and Co in Liusha Bay’s mangrove sediments may mainly stem from differences in regional geological backgrounds—the sediments in this region are more affected by local volcanic rock weathering materials rich in Cu, Cr, Ni, and Co [33], while intensive mariculture may contribute to the concentrations of Cu and As in Liusha Bay’s sediments [41]. In contrast, the high concentrations of Cu, Cr, Ni, and As in mangrove sediments of the Pearl River Delta may originate from wastewater rich in Cu, Cr, Ni, and As discharged by the region’s developed industries [52].

The Zn concentration in Liusha Bay’s mangrove sediments is significantly lower than that in mangrove sediments of the economically developed Pearl River Delta and Fujian Province, and equivalent to that in mangrove sediments of the Guangxi Zhuang Autonomous Region and Hainan Province (with similar economic development levels); the Pb and Cd concentrations are at relatively low levels among mangrove sediments of the above-mentioned provinces in China. Specifically, regarding pollution levels, the I_geo_ values of Pb, Zn, and Cd in Liusha Bay’s mangrove sediments are mostly less than 0, basically unpolluted; Pb and Zn in mangrove sediments of the Pearl River Delta and Fujian Province are at light to moderate pollution levels, while Cd pollution ranges from light to extremely high levels.

In terms of ecological risk, the overall ecological risk levels of Pb and Zn in sediments of China’s mangrove regions are relatively low; Cd in some stations of Liusha Bay’s mangrove sediments is at the “moderate risk” level, while Cd in mangrove regions of Fujian Province, the Pearl River Delta, and parts of the Guangxi Zhuang Autonomous Region is at levels ranging from “moderate risk” to “extremely high risk”.

The low concentrations and low pollution levels of Pb, Zn, and Cd in Liusha Bay’s mangrove sediments may mainly be attributed to the relatively low industrial pollution load and low natural background values of these elements in this region [15]. Studies have shown that the background values of Pb, Zn, and Cd in the soil of the Leizhou Peninsula are 20 mg·kg^−1^, 51.8 mg·kg^−1^, and 0.041 mg·kg^−1^, respectively [33]. In contrast, Cd pollution in mangrove sediments of the Pearl River Delta and Fujian Province may mainly originate from industrial pollution [52].

### 4.2. Sources of Heavy Metals

The results of principal component analysis (PCA) indicate that major elements and trace elements in mangrove sediments of Liusha Bay can be divided into four principal components (PCs), with the cumulative variance contribution rate of the first three PCs being approximately 90%. Similarly, hierarchical cluster analysis shows that major and trace elements in sediments of the study area can be classified into three major element groups, which are further subdivided into seven subgroups. There is a correspondence between the cluster analysis results and the PCA: Element Group I corresponds to PC1, Element Group III corresponds to PC2, Element Subgroup II_1_ is close to the characteristics of PC3, and Element Subgroup II_2_ is close to the characteristics of PC4.

#### 4.2.1. Element Group I (Corresponding to PC1, Variance Contribution Rate ~50%)

This element group can be further subdivided into two subgroups:

- Element Subgroup I_1_ (Ti, Fe, Na, Zn, V, Sc, Al, Zr): Ti, Al, Zr, and Sc are typical indicators of terrigenous detritus. For example, Zr is mainly hosted in heavy minerals (e.g., zircon) and forms detritus through transport and deposition; Al is widely present in clay minerals. These terrigenous elements accumulate in coastal sediments through geological processes such as weathering, erosion, transport, and deposition [49,50].
- Element Subgroup I_2_ (Mg, As, Cr, Ni, Co): Mg, Cr, and Ni are mainly derived from the erosion and release of magmatic rocks such as basalt and ultramafic rocks [53,54]. Heavy metal speciation analysis shows that Zn, Cr, As, and Ni are dominated by the residual fraction (accounting for over 70% each), indicating that these four elements are mainly hosted in geochemically stable terrigenous minerals.

Previous studies have shown that mangrove sediments in northern Hainan Island are dominated by terrigenous detritus, with their main factors including elements such as Al, Fe, and Ti—these elements have stable geochemical properties and are hosted in clay minerals and fine-grained terrigenous detritus [44]. In mangrove sediments of northern Leizhou Bay (Zhanjiang Bay), Co is mainly derived from the weathering of soil parent materials, while Cr is partially from parent materials and partially affected by coastal domestic sewage [41]. For mangrove sediments in Nanshan Town, Xuwen (also in the southern Leizhou Peninsula), Ni pollution was once thought to be caused by domestic waste or wastewater (e.g., waste Ni batteries) [39]. However, in this study area, there is a significant correlation among Cr, Ni, and Co, and three discretely distributed sampling stations all show similar characteristics, suggesting a more widespread pollution source (rather than occasional pollution from waste batteries).

In addition, mangrove areas in Donghai Island (northern Leizhou Peninsula) are affected by aquaculture, and heavy metals such as As, Cr, Cu, Ni, Pb, and Zn in sediments are considered closely related to aquaculture discharge [38]; similar phenomena have also been observed in mangrove areas of Zhanjiang Bay [41], Hainan Island [55], and Fujian Province [56]. It is thus inferred that high-density aquaculture in Liusha Bay may partially affect the concentrations of heavy metals such as As, Cr, Ni, and Zn in the study area.

Considering the regional geological background, Quaternary volcanic rocks are widely distributed in the southern Leizhou Peninsula where Liusha Bay is located (the lithology is dominated by dark gray-yellowish brown basaltic tuff, agglomerate, and basalt), and the parent material of terrestrial soils is mainly weathered volcanic rocks. Compared with weathered sedimentary rocks, weathered volcanic rocks are enriched in elements such as Cu, Cr, Ni, and Co (with concentrations over three times those in weathered sedimentary rocks; [33])—mangrove sediments in Nanshan Town, Xuwen (also in the southern Leizhou Peninsula) also show high concentrations of Cu, Cr, Ni, and Co, which can serve as evidence. Notably, previous comparisons show that the concentrations and pollution levels of Cu, Cr, Ni, and Co in Liusha Bay sediments are relatively high among mangrove areas in China; however, with little industrial pollution and sparse population in this region, these elements are likely dominated by natural background.

In summary, Element Group I represents terrigenous materials: Fe, Zn, Zr, etc., in sediments of the study area are mainly affected by fine-grained terrigenous materials; As, Cr, Ni, and Co are mainly affected by terrigenous weathered volcanic rocks, while As, Cr, Ni, and Zn may be partially affected by aquaculture discharge.

#### 4.2.2. Element Group III (Corresponding to PC2, Variance Contribution Rate ~30%)

This element group is subdivided into three subgroups, characterized by calcareous biological sources:

- Element Subgroup III_1_ (Ca, Sr): Ca is a characteristic element of marine-derived sediments, representing calcareous biological deposition; Sr is a biophilic element, often enriched in biological shells and debris.
- Element Subgroup III_2_ (Cu, P, Mn, Cd): These elements, together with Ca and Sr, belong to the biological source group—Cu, P, and Mn are essential biological elements, while Cd is non-essential; all may originate from the enrichment and redeposition processes of calcareous organisms [57,58]. Pearson correlation analysis shows that Cu is significantly correlated with Ca, Cd, Mn, Sr, and P, further confirming the consistency of their sources. In heavy metal speciation analysis, the carbonate-bound fraction of Cu accounts for 9% and the organic-bound fraction accounts for 31% (both the highest among all heavy metals), also indicating its biological source characteristics.
- Element Subgroup III_3_ (Ba only): Ba also has biological source characteristics [58].
- Mangrove areas in northern Hainan Island also have biological deposition dominated by Ca and P [44]. Previous surveys on bivalves and benthic gastropods in Liusha Bay show significant Cd enrichment in these organisms, indicating Cd pollution in Liusha Bay [59]. Although there are few industrial enterprises around the study area, shipping and related operations are frequent—ship paints contain heavy metals such as Cu, Zn, Pb, and Cd; after paint peeling and release, these metals may be enriched by calcareous organisms and deposited, thereby causing pollution [9,42].

In summary, Element Group III mainly represents calcareous biological sources: Cu, Mn, and Cd are mainly affected by calcareous biological materials, while Cu and Cd may be additionally affected by heavy metals released from ship paints.

#### 4.2.3. Element Group II (Including K, Rb, Pb, Th, S, U)

Among these elements, K, Rb, and Pb are lithophile elements [60] (commonly found in potassium feldspar); U and Th are common in magmatic rock minerals [61]; S is widely distributed, mainly in the form of sulfides or sulfates [62].

In mangrove sediments of Zhanjiang Bay (northern Leizhou Peninsula), Pb pollution is considered to originate from industrial lead emissions, agricultural use of lead-containing pesticides and fertilizers, and aquacultural application of lead-containing fish feed [41]. There are few industries around Liusha Bay, but Pb pollution from agriculture and aquaculture cannot be ignored (current pollution levels are generally low).

In summary, Element Group II may originate from terrigenous weathered materials such as potassium feldspar, among which Pb, Th, and U are mainly affected by such terrigenous weathered materials (Note: Th and U are radioactive elements, not traditional heavy metals, and are analyzed here uniformly according to element group characteristics).

## 5. Conclusions

This study analyzed heavy metal characteristics in mangrove sediments of Liusha Bay, Leizhou Peninsula, China. Key findings: Arsenic (As, all sites polluted) and copper (Cu, some moderately polluted) were main pollutants; nickel (Ni), chromium (Cr), cobalt (Co) showed light pollution; lead (Pb), zinc (Zn), cadmium (Cd) caused no pollution. Only individual sites had moderate ecological risk from As/Cd, with overall low risk. Except Cd (mainly exchangeable, high bioavailability), most heavy metals were in low-risk residual fractions. They primarily came from terrigenous sources (volcanic rock weathering, detritus) and calcareous organisms, with minimal impacts from aquaculture or ship coatings. Compared to other regions, this area had significantly higher Ni/Cr and lower Pb/Zn/Cd, linked to a volcanic geological background and low industrial pollution.

Future research can be advanced in three aspects: first, conduct long-term seasonal monitoring to clarify the dynamic changes in heavy metal contents; second, quantify the contribution ratio of human activities (e.g., aquaculture, ship coatings) to heavy metals; and third, explore the accumulation effect of highly exchangeable Cd on mangrove organisms and the release and migration paths of heavy metals during volcanic rock weathering, so as to provide more precise support for regional ecological protection.

## Figures and Tables

**Table 1:** Heavy metal pollution and ecological risk assessment methods.

Pollution Indicators	Calculation Formula and Classification Level					
Geo-accumulation index (I_geo_) [21,22]	$I_{geo}={log}_{2}(\frac{C_{n}}{1.5B_{n}})$					
	C_n_ is the measured concentration of the target element in sediments; B_n_ is the environmental background value of the element, and the average upper-crust element concentration was selected in this study [23]. I_geo_ can be divided into six grades:					
	I_geo_ ≤ 0	0 < I_geo_ ≤ 1	1 < I_geo_ ≤ 2	2 < I_geo_ ≤ 3	3 < I_geo_ ≤ 4	I_geo_ > 4
	Uncontaminated	Slightly	Moderately	Moderately/Heavily	Heavily	Extremely
Enrichment factor (EF) [22,24]	$EF=\frac{{\left(C_{i}/C_{r}\right)}_{sample}}{{\left(C_{i}/C_{r}\right)}_{background}}$					
	(C_i_/C_r_) sample and (C_i_/C_r_) background are the concentration ratios of the target element to the reference element in sediment samples and background values, respectively; the average upper-crust element concentration was used as the background value [23]; C_i_ is the concentration of target element i, and C_r_ is the concentration of the reference element. Al was selected as the reference element in this study due to its wide distribution in the earth’s surface, stable chemical properties, low volatility, and minimal interference from human activities. EF can be divided into six grades:					
	EF ≤ 1	1 < EF ≤ 2	2 < EF ≤ 5	5 < EF ≤ 20	20 < EF ≤ 40	EF > 40
	Uncontaminated	Slightly	Moderately	Moderately/Heavily	Heavily	Extremely
Ecological risk assessment ($E_{r}^{i}$) [25,26]	$E_{r}^{i}=\frac{T_{r}^{i}C_{r}^{i}}{C_{n}^{i}}$					
	$T_{r}^{i}\;$ is the toxicity coefficient of heavy metal i; $C_{r}^{i}$ is the concentration of element i in the sediments to be assessed; $C_{n}^{i}$ is the concentration of element i in background sediments, and the average upper-crust element concentration was selected as the background value in this study [23]. $E_{r}^{i}$ can be divided into five grades:					
	$E_{r}^{i}$ < 40	40 ≤ $E_{r}^{i}$ < 80	80 ≤ $E_{r}^{i}$ < 160	160 ≤ $E_{r}^{i}$ < 320	$E_{r}^{i}$ > 320	
	Low risk	Moderate risk	Considerable risk	High risk	Extremely high risk	

**Table 2:** Concentration of major elements in sediments (%).

Station	Ti	Al	Fe	Mg	Ca	Na	K	S
LS1	0.67	7.04	6.49	1.22	1.53	1.26	0.72	1.25
LS2	0.49	4.36	5.04	0.54	1.82	0.65	0.25	0.69
LS3	0.98	7.6	6.88	0.89	1.22	1.65	0.61	0.43
LS4	0.57	4.58	4.02	0.27	0.12	0.43	0.62	0.09
LS5	0.73	8.21	5.98	0.52	0.38	1.07	0.93	1.52
LS6	0.52	4.66	4.96	0.84	6.63	0.87	0.4	0.75
Mean	0.66	6.08	5.56	0.71	1.95	0.99	0.59	0.79
S.D.	0.18	1.73	1.08	0.34	2.39	0.44	0.24	0.52
MAX	0.98	8.21	6.88	1.22	6.63	1.65	0.93	1.52
Min	0.49	4.36	4.02	0.27	0.12	0.43	0.25	0.09
upper-crust element concentration [22]	0.38	8.15	3.92	1.5	2.57	2.43	2.32	0.062

## Data Availability

The original contributions presented in this study are included in the article. Further inquiries can be directed to the corresponding authors.

## Appendix A

Concentrations of major and trace elements in mangrove sediments of Liusha Bay.

## Appendix B

The spatial distribution of heavy metal element concentration, geo-accumulation index, enrichment factor and ecological risk index of mangrove sediments in Liusha Bay.

## Appendix C

Comparative maps of heavy metal concentration and pollution status in sediments of Liusha Bay, Leizhou Peninsula offshore waters and mangrove forests in China.
